# Context-Specific Proportion Congruency Effects: An Episodic Learning Account and Computational Model

**DOI:** 10.3389/fpsyg.2016.01806

**Published:** 2016-11-16

**Authors:** James R. Schmidt

**Affiliations:** Department of Experimental Clinical and Health Psychology, Ghent UniversityGent, Belgium

**Keywords:** context-specificity, contingency learning, temporal learning, computational modeling, attention, conflict monitoring, proportion congruent effect, Stroop task

## Abstract

In the Stroop task, participants identify the print color of color words. The congruency effect is the observation that response times and errors are increased when the word and color are incongruent (e.g., the word “red” in green ink) relative to when they are congruent (e.g., “red” in red). The proportion congruent (PC) effect is the finding that congruency effects are reduced when trials are mostly incongruent rather than mostly congruent. This PC effect can be context-specific. For instance, if trials are mostly incongruent when presented in one location and mostly congruent when presented in another location, the congruency effect is smaller for the former location. Typically, PC effects are interpreted in terms of strategic control of attention in response to conflict, termed conflict adaptation or conflict monitoring. In the present manuscript, however, an episodic learning account is presented for context-specific proportion congruent (CSPC) effects. In particular, it is argued that context-specific contingency learning can explain part of the effect, and context-specific rhythmic responding can explain the rest. Both contingency-based and temporal-based learning can parsimoniously be conceptualized within an episodic learning framework. An adaptation of the Parallel Episodic Processing model is presented. This model successfully simulates CSPC effects, both for contingency-biased and contingency-unbiased (transfer) items. The same fixed-parameter model can explain a range of other findings from the learning, timing, binding, practice, and attentional control domains.

## Introduction

One of the main areas of interest in experimental psychology is how the cognitive system controls attention to maximize task performance (Treisman and Gelade, 1980; Miller and Cohen, 2001; Petersen and Posner, 2012). Consider the classic Stroop task (Stroop, 1935). Participants are much slower and more error prone to identify the color of color words on *incongruent trials* (e.g., the word “red” printed in blue) relative to *congruent trials* (e.g., “red” in red). The difference in performance between these two conditions is termed the *congruency effect*. Of course, the impairments on incongruent trials indicate a (partial) failure of selective attention. However, participants are reasonably accurate, indicating that participants are able to instantiate the goal to ignore the word and attend to the print color. In addition to strategic adjustments of attention at the start of an experiment in order to achieve task goals, the cognitive system might continue to adapt attentional control dynamically throughout a task. One particularly impactful idea, called the *conflict monitoring (or conflict adaptation) account*, is that attention is dynamically adjusted on a trial-by-trial basis in reaction to response conflict (Botvinick et al., 2001).

One of the main sources of evidence presented in support of the conflict monitoring account is the *proportion congruent (PC) effect* (Logan and Zbrodoff, 1979; Lowe and Mitterer, 1982; Logan et al., 1984). The PC effect is the observation that congruency effects are much diminished when trials are *mostly incongruent* (e.g., 75% incongruent, 25% congruent) relative to *mostly congruent* (e.g., 75% congruent, 25% incongruent). According to the conflict monitoring account, participants adapt to the frequent conflict in the mostly incongruent condition by decreasing attention to the distracting word (and/or increasing attention to the color). As such, the word has a reduced impact on color identification, thereby diminishing the congruency effect. In the mostly congruent condition, however, conflict is less frequent, and attention is, as a result, more “lazy.”

Though extremely popular, the conflict monitoring account has been contested on several fronts (for reviews, Schmidt, 2013b; Schmidt et al., 2015). For instance, Schmidt and Besner (2008; see also, Atalay and Misirlisoy, 2012; Grandjean et al., 2013; Schmidt, 2013a, 2014a, 2016a; Hazeltine and Mordkoff, 2014; Levin and Tzelgov, 2016) presented the argument that the (item-specific) PC effect might be due (primarily or even entirely) to the learning of contingencies (i.e., correlations) between color words and responses (for related ideas, see Logan et al., 1984; Mordkoff, 1996; Melara and Algom, 2003). For instance, in the mostly congruent condition, words are presented most often in their congruent color (e.g., “red” 75% of the time in red). As such, color words are strongly predictive of the congruent response, which benefits congruent trials. On incongruent trials (e.g., “red” in green), however, the word mispredicts the color response, resulting in a cost. The net result is an increased congruency effect. In the mostly incongruent condition, the situation is reversed. Depending on the exact manipulation, color words might be presented most often in a specific incongruent color (e.g., “green” most often in red, etc.). Thus, words are accurately predictive of the *incongruent* response, and mispredict a congruent response. The net effect is a reduced congruency effect. What is most interesting about the contingency learning account of the PC effect is that it is unrelated to conflict, control, or attention. The account argues that the learning of stimulus-response correspondences is all that matters.

Currently, it is general consensus that the majority of the PC effect in the prototypical paradigm is due to contingency learning. However, it is also clear that there are findings that cannot be explained by simple contingency learning, and debate still continues as to whether conflict monitoring produces these remaining components of the PC effect (Blais and Bunge, 2010; Bugg and Chanani, 2011; Bugg et al., 2011a,b; Atalay and Misirlisoy, 2012; Bugg and Hutchison, 2013; Grandjean et al., 2013; Schmidt, 2013a,c, 2014a,b, 2016a; Bugg, 2014, 2015; Hazeltine and Mordkoff, 2014; Levin and Tzelgov, 2016). For instance, consider the list-level PC effect. Some *inducer items* are manipulated for PC in separate groups of participants (e.g., “red” and “blue” mostly congruent for one group, and mostly incongruent for another), and these are intermixed with other (contingency-unbiased) *diagnostic items* (e.g., “green” and “yellow,” which have the same PC for both groups). A (list-level) PC effect is, in some cases, observed for the diagnostic items (e.g., Hutchison, 2011; Bugg, 2014; Wühr et al., 2015; cf., Bugg et al., 2008; Blais and Bunge, 2010). That is, the PC effect of the inducer items generalizes to the diagnostic items.

**FIGURE 1:**
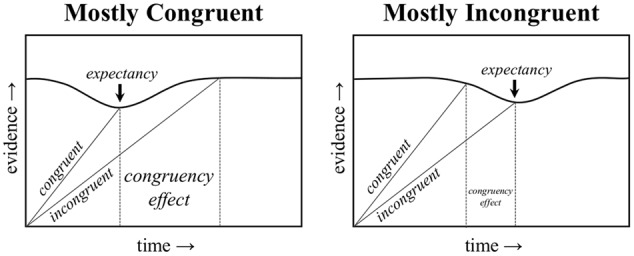
**A representation of how expectancy-driven reductions in the response threshold can produce a proportion congruent effect.** An earlier drop in the threshold in the mostly congruent condition benefits congruent trials, whereas a later drop in the threshold in the mostly incongruent condition benefits incongruent trials.

Obviously, list-level PC effects cannot be explained by contingency learning, given the lack of a contingency for diagnostic items. However, Schmidt (2013c, 2014b) argued that the list-level PC effect could be explained by temporal learning (i.e., rhythmic responding). Response times tend to be rhythmic, with the current RT typically similar to the RTs of immediately preceding trials (Grosjean et al., 2001). This has implications for (list-level) PC effects, because the task “pace” will tend to be faster in the mostly congruent condition (where most trials are easy) relative to the mostly incongruent condition (where most trials are hard). According to the temporal learning account, response initiation is speeded when the task pace can be maintained. In particular, it is proposed that the (otherwise stable) *threshold* to respond is decreased at the expected time, as illustrated in **Figure 1**. That is, the amount of evidence needed to select a response temporarily decreases when the expected time approaches. If activation (evidence) for a response is sufficient to cross this reduced threshold, responding is speeded (i.e., because less evidence accrual was required to determine the response) and the task pace is maintained. In contrast, if insufficient activation has accrued to determine a response at the expected time (e.g., because the trial was harder than average), then the task pace is “broken,” the threshold to respond increases back to normal, and the rhythmic benefit is lost. Similarly, if a response is determined *before* the expected time to respond (e.g., because the trial was easier than average), responding will, of course, be fast, but will not have benefitted from a reduced threshold to respond.

**FIGURE 2:**
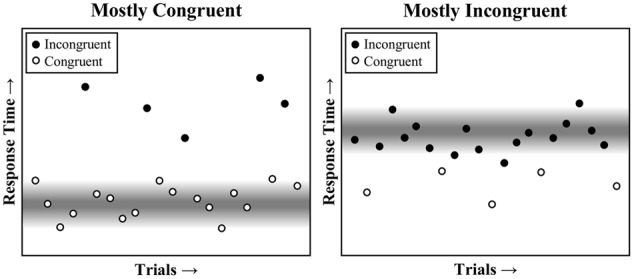
**A visual representation of task pace on performance across a series of trials in the mostly congruent and mostly incongruent conditions.** The response threshold is diminished at the expected time to respond (gray), speeding any trials that can benefit from this reduced threshold. This primarily benefits congruent trials in the mostly congruent condition and incongruent trials in the mostly incongruent condition.

Because the mostly congruent condition contains mostly easy, and therefore fast, congruent trials, the expected time to respond will be earlier in the trial (i.e., around the time of a typical congruent trial). As such, the faster task pace in the mostly congruent condition will tend to benefit congruent trials, which fit into the task rhythm, but not incongruent trials, which break the task rhythm. The reverse is true in the mostly incongruent condition, where a slower pace benefits incongruent trials, but not congruent trials. This is also illustrated in **Figure 1**, and is further illustrated visually in a trial-by-trial representation in **Figure 2**. Temporal learning may therefore account for list-level PC effects (Kinoshita et al., 2011), even for contingency-unbiased diagnostic items (Schmidt, 2013c, 2014b, 2016b). As an added aside, some threshold adjustment mechanisms can produce a speed-accuracy trade-off, such that responding is either faster but more error prone (low threshold) or slower but less error prone (high threshold). However, the dynamically adjusting threshold in the Parallel Episodic Processing (PEP) model does not produce a speed-accuracy trade-off in PC (or congruency sequence) effects, but instead produces the same pattern in errors as in response times (Schmidt, 2013c; Schmidt and Weissman, 2016), just as in the participant data. This is because the threshold is fixed, only temporarily dipping early in the mostly congruent condition (benefitting congruent, but increasing fast incongruent errors) and the reverse in the mostly incongruent condition.

The above discussion illustrates how contingency and temporal learning processes might explain item-specific and list-level PC effects. The present manuscript focuses on another variant of the PC task. The *context-specific proportion congruent (CSPC) effect* is the finding that a PC effect is still observed when mostly congruent and mostly incongruent trials are randomly intermixed and only distinguished by a secondary contextual cue. For instance, Corballis and Gratton (2003; see also, Wendt et al., 2008) used flanker (rather than Stroop) stimuli and presented participants mostly congruent stimuli on one side of the screen (e.g., left), and mostly incongruent stimuli on the other side (e.g., right). Even though (a) the two context were randomly intermixed, and (b) the exact same stimuli were used in both locations, congruency effects were smaller in the mostly incongruent location than in the mostly congruent location. In subsequent reports, Crump et al. (2006, 2008) used a similar manipulation with Stroop-like stimuli. Again, a CSPC effect was observed. Other reports have used stimulus dimensions other than location as the contextual cue, such as text font (Bugg et al., 2008), time duration (Wendt and Kiesel, 2011), and color (Lehle and Hubner, 2008), but the present discussion will be framed in the location-specific Stroop manipulations of Crump and colleagues.

Typically, CSPC effects are interpreted in terms of context-specific conflict monitoring (e.g., Crump et al., 2006). The notion is that attention is more stringently controlled in the mostly incongruent context (e.g., location), and more lax in the mostly congruent context (e.g., another location). This, of course, implies that attentional control can be adjusted extremely rapidly: before a trial begins, the participant cannot possibly know which contextual location they will experience. Indeed, a particularly interesting detail of the experiments by Crump et al. (2006, 2008) is that color words were presented at fixation, then removed from the screen before a color patch was presented either above or below fixation. Thus, participants could not know the PC context until *after* the word was already removed from the screen (i.e., when target location was known). As such, participants cannot know whether or not to attend to the word until after it is already gone. This clearly indicates that if attentional control is involved, it cannot be control of attention to perceptual inputs, but must be occurring somewhere further down the processing stream.

It is noteworthy that, task wide, words are not predictive of colors in CSPC designs. Contingency learning may nevertheless account for (part) of the CSPC effect, because the word and location combined do strongly predict the color response. For instance, if the mostly congruent location is up, then “red” plus “up” likely indicates a red response. In contrast, “red” plus “down” likely indicates a green response (or no response, depending on the manipulation). In other words, the contingency bias is identical, save for the fact that word and location information must be used jointly to predict the color response. Learning based on feature conjunctions is known to occur outside the context of conflict paradigms like the Stroop task (Mordkoff and Halterman, 2008), so it seems reasonable that location-word combinations might be used to predict the likely color response (see also, Holland, 1992 for a review of occasion setting).

**FIGURE 3:**
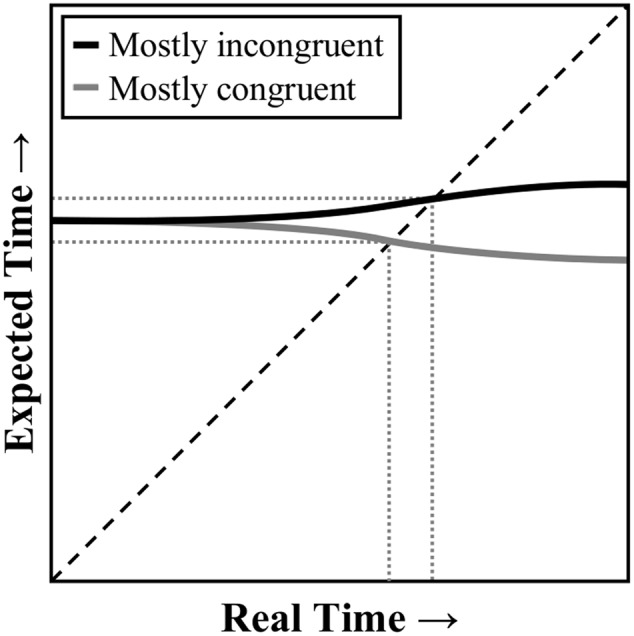
**Illustration of a temporal learning mechanism as it relates to context-specific proportion congruent effects.** At the start of the trial, the expected time to respond is the same for a mostly congruent or mostly incongruent stimulus. However, as the contextual stimulus (e.g., location) is processed, the expected time to respond can adjust dynamically. The response threshold is decreased when real time is close to the expected time to respond. Dashed line represents where expected time equals real time. Dotted lines indicate the points where the current expectancy (solid line) intersects with current real time.

However, context-specific contingency learning cannot be the whole story. Crump and Milliken (2009; see also, Heinemann et al., 2009; Crump et al., 2016; but see, Hutcheon and Spieler, 2016) further demonstrated that if CSPC is manipulated with some (contingency-biased) inducer items, there is still a CSPC effect for other non-manipulated (contingency-unbiased) diagnostic items. Paralleling the above discussion of item-specific and list-level PC effects, the context-level (i.e., non-item-specific) component of the CSPC effect might be explained by context-specific (i.e., location-specific) temporal learning. The only added assumption would, again, be that (non-conflict) learning can be context-specific. In this case, participants might be learning a different pace for each contextual location. In support of this notion, Schmidt et al. (2014) have already demonstrated that a CSPC-like interaction can be observed in a non-conflict task. In particular, “mostly easy” and “mostly hard” location contexts were created with “easy” and “hard” items of a completely different sort: high contrast (i.e., easy to see) and low contrast (i.e., hard to see) letters. **Figure 3** illustrates how context-specific temporal learning can come about. At the start of the trial, of course, the participant cannot know which location context they will experience. However, as soon as the target color appears above or below fixation, knowledge of the stimulus location can be used to rapidly adjust the expected time to respond. Thus, an earlier expected time to respond in the mostly congruent than mostly incongruent condition can again be determined.

As the above discussions illustrate, the same procedural concerns in standard, item-specific, and list-level PC designs also apply to the CSPC design. The only added assumption is that learning (of both contingencies and temporal information) can be context/location-specific. Indeed, as the following sections will demonstrate, this “added” assumption is not much of an assumption at all, because context-specificity can be a simple by-product of the same memory mechanisms required for simple item-specific learning. In the simulations to follow, it will be demonstrated how the PEP model is able to simulate CSPC effects, including for contingency-unbiased diagnostic items, using only contingency and temporal learning mechanisms.

## Materials and Methods

### The Model

In this section, a brief conceptual overview of the PEP model is presented. A full description of the math of Version 2.0 of the model can be found in Schmidt et al. (2016), and the one small change made for the current Version 2.1 of the model can be found in the Supplementary Material of the current report. Full documented source code of the model can also be downloaded from the website of the author ^1^.

**FIGURE 4:**
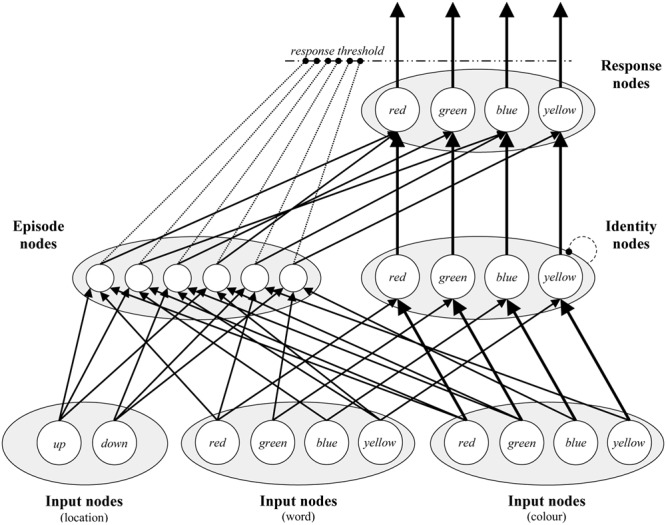
**A visual depiction of the Parallel Episodic Processing (PEP) model.** There are Input nodes for each location, word, and color. Input nodes feed activation to Identity nodes, where conflict can occur between color words and colors. Identity nodes feed activation to Response nodes, where a response is selected. Via Episode nodes, locations, words, and colors activate the responses that they are connected to, in addition to affecting the response threshold dynamically. Connections ending in circles indicate inhibition.

The PEP model is depicted visually in **Figure 4**. At the bottom of the model, there are Input nodes for each stimulus type (i.e., colors, words, and locations). On each trial, all Input nodes are activated with random noise, and presented stimuli are further activated with a signal. Thus, the model begins each trial with random biases for each input, but over time identifies the color, word, and location that are presented on the trial. Word and color Input nodes pass on activation to Identity nodes, one for each color concept. Within these nodes, lateral inhibition produces a main effect of congruency. Note that locations do not connect to Identity nodes, because they are unrelated to the target color dimension (this is also true of words when simulating experiments with color-unrelated neutral words), but location nodes are otherwise exactly identical to word nodes. Finally, activation from Identity nodes feeds forward to Response nodes. Once activation for one of the Response nodes exceeds the response threshold, the model responds. The number of “cycles” (simulated milliseconds) the model takes to respond is the simulated response time.

The above describes the algorithmic route of the model. The actual “work” of the model in producing the effects of interest in PEP model simulations is in the Episode nodes. During each trial, a new Episode node is created, which codes for the stimuli presented (word, color, and location), the response that was made, and also the response time. On subsequent trials, episodes are retrieved on the basis of the stimuli being currently presented to the model. For instance, the word “blue” will activate episodes coding for the word “blue.” Activated episodes have two effects on processing. The first is via the contingency learning mechanism (Schmidt, 2013a, 2016a; Schmidt et al., 2016). Each activated episode facilitates response nodes proportional to (a) how active the Episode node is, and (b) how strong the connection between the episode and response is. This produces a benefit for high contingency (accurately predictive) over low contingency (wrongly predictive) trials, because, for instance, if “green” is presented most often in red, then presenting the word “green” will activate “green”-word episodes, most of which code for (and therefore facilitate) red responses. Though less relevant for the current simulations, recently encoded episodes have a stronger effect on retrieval than older ones (for an extended discussion, see Schmidt et al., 2016).

The second way that episodic retrieval influences processing is via the temporal learning mechanism (see Schmidt, 2013c; Schmidt and Weissman, 2016). In particular, activated episodes are used to determine the expected time to respond. Like with the contingency learning mechanism, recent events have a much stronger effect on current-trial expectancies. As such, the model expects to be able to respond at a similar speed as on previous trials (see the Supplementary Material for the math on how this is computed). The response threshold (i.e., amount of activation required to select a response) temporarily decreases around the time at which the current cycle time is close to the expected time to respond. In this way, responding is speeded if the “pace” can be maintained (i.e., a response is able to cross the temporarily decreased threshold), but is not speeded if the pace is broken (e.g., not enough evidence for a response at the expected time).

### Model Changes

No modifications need to be made to the PEP model to simulate context-specific contingency learning. Context-specific contingency learning is a logical consequence of the same learning mechanism used for regular (“item-specific”) contingency learning. With a “context-specific” PC manipulation all that has changed is an added feature (e.g., location). If “red” is presented most often in green in the bottom location, then when “red” and “down” are presented together “red” will activate “red” episodes and “down” will activate “down” episodes. This means that episodes encoding for *both* “red” and “down” will be very strongly activated. These episodes, of course, predominately point to a green response. Thus, according to this episodic conceptualization, “item-specific” and “context-specific” learning are not really different. The only minor difference between the two is that “context-specific” learning involves more than one predictive dimension.

Changes to the temporal learning mechanism, however, *were* required to simulate context-specific temporal learning. In the previous versions of the PEP model, the temporal learning mechanism was relatively simple. After each trial, the response time was (and still is) directly encoded into the episode for that trial. During each trial, the model retrieved the last five encoded episodes and each of these episodes decreased the response threshold at the encoded time. The most recent episode had the largest impact on the threshold and older ones had less and less impact. One thing to note about this instantiation of a rhythmic responding mechanism is that it is not item-specific. The last five episodes are retrieved regardless of whether these episodes match the currently presented stimuli. Secondly, episodes are retrieved at a strength directly proportional to how old they are and not based on connection weightings and episode activations. This formulation of the temporal learning mechanism was for simplicity only and changes in Version 2.1 of the PEP.

In the new version of the temporal learning mechanism, several key changes are made. First, a single *pace* (expected time to respond) is computed on each trial, rather than each retrieved episode affecting the response threshold separately. Second, the pace is adjusted during the course of the trial on the basis of which episodes are activated. Thus, if the episodes that are activated all happen to be trials in which a fast response was made, the pace will speed. Conversely, if episodes where slow responses were made are retrieved, the pace will slow. Thus, item-specific (and context-specific) influences on the current-trial rhythm are possible. Third, how much influence a given episode has on the pace is determined by the weighting of the time information for that episode. Just like the connections between episodes and stimuli or responses, time weightings (newly added to the model) are weakened with retrieval. Thus, recent events still have much larger impacts on the response threshold, but for a different reason than in the previous version of the model. Note that this change also makes the contingency and temporal learning mechanisms similar to one another (e.g., the prediction of *what* response to make and *when* to make it are both determined by the same episodic activations).

Full details of the new code are presented in the Supplementary Material, but here a walkthrough is presented as to how the temporal learning mechanism can produce a CSPC effect for contingency-unbiased transfer items. At the start of a trial, the pace is determined by the previous trial pace and the immediately preceding RT. As such, the model is blind to the upcoming context at this point. As the model begins to retrieve episodes, however, the pace will adjust. If, for instance, a color stimulus is presented in the top of the screen, episodes encoding for “up” will become more active than episodes encoding for “down.” If mostly congruent stimuli have been presented in the top location, then most of these episodes will have a fast RT encoded. As such, these activated episodes will bias the pace toward a faster response. That is, the model expects a response earlier on in the trial, which works to the advantage of (normally fast) congruent trials, thereby increasing the congruency effect. In contrast, when the color stimulus is presented in the mostly incongruent location, most of the retrieved episodes will point to a slow response. As such, a response will be expected later in the trial, and this will work to the advantage of incongruent trials, rather than congruent trials.

Because it may be unclear, it is important to stress why this works for *contingency-unbiased* diagnostic items. Of course, episodes that *exactly* match the current-trial stimuli (i.e., same word, same location, and same color) are the most strongly retrieved. However, episodes that only *partially* match the current-trial stimuli are also retrieved to a lesser extent. For instance, a diagnostic stimulus such as “blue” in yellow in the top location will partially activate all nodes that code for the word “blue,” all nodes that code for the color yellow, and, more crucially, all nodes that code for the top location. Thus, “blue” in yellow in the top location will also (partially) activate the *inducer* items for the top location (e.g., “red” in red), which will be 100% congruent. This is similar to the MINERVA 2 model of Hintzman (1986), where multiple episodes are retrieved in parallel, each in proportion to the overlap between the stimulus presented and the stimulus encoded in memory (i.e., such that partially similar episodes provide a weak contribution to the “echo” from memory, and highly similar episodes provide a stronger contribution). Thus, even though episodes are encoded in a 100% item-specific fashion, retrieval from memory does produce generalization. That is, inducers will influence time expectancies for diagnostic items in a context-specific fashion.

### Simulation Procedure and Data Analysis

All simulations were run with one fixed parameterization. That is, the only thing that changes from simulation to simulation is which stimuli are presented to the model. Using the same fixed parameterization, it was also confirmed that Version 2.1 of the PEP model is backward compatible with previous simulations (i.e., the model still produces all the same effects that it was previously reported to simulate). Note that the model aims for rough qualitative fit to data and does not aim to match effects (or even overall RT) cycle-for-millisecond. This is particularly the case given that the same fixed-parameter model is used to simulate a broad range of effects from drastically different paradigms (e.g., verbal and keypress Stroop, single letter identification, prime-probe arrow tasks, Eriksen flanker tasks, etc.). That is, rather than overfitting a model to one or two specific experiments, the PEP model aims to demonstrate whether or not a given process is able to produce a range of different effects. Thus, the aim of the present simulations is to determine whether the processes instantiated in the model are sufficient to produce a CSPC effect of the correct form, and not to match exact effect sizes.

Note that each simulated “participant” has the same base parameters (e.g., same learning rate, etc.). This might be reasonably changed in the future to model inter-individual differences (e.g., by assigning parameters to each participant from a Gaussian distribution). However, there is plenty of noise throughout the model, such that each simulated participant does produce different results. To ensure robustness, 500 simulated “participants” were run for each simulation. Given this large “sample,” certainty of whether the model does or does not produce an effect is well above that for a typical experimental sample (i.e., *p*s ≪ 0.05). Thus, numerical effect sizes are discussed, but no statistics are reported for brevity. However, all effects that are discussed are statistically significant. Also, only response times are presented both for brevity and because the original CSPC experiments reported null CSPC effects for errors (or did not analyze errors at all). However, results for errors are comparable to the RT data (i.e., there is no speed-accuracy trade-off; see “Introduction”) and are not excessively inflated (as can be a problem in some models). It is worth noting, however, that errors were essentially non-existent (<1%) in the original participant data, whereas the model produced incongruent trial error rates more comparable to a standard Stroop paradigm. The smaller error rates in the CSPC participant data relative to a standard Stroop task might be due to the spatially- and temporally-separated distracters, a detail which is not reflected in the PEP model. This point will be discussed in further detail in the “General Discussion.” Error data and the data from the backward compatibility simulations are available for download from the website of the author (along with the model code download).

### Simulation 1: Base CSPC Effect

Simulation 1 models the basic location-based CSPC effect. In particular, Experiment 2a of Crump et al. (2006) is simulated. The design is presented in **Table 1**. Trials consist of the word “red,” “green,” “blue,” or “yellow” presented at fixation for 100 cycles followed by the a red, green, blue, or yellow patch presented above or below fixation until a response or 2000 cycles elapsed. In each block of 96 trials, there are 48 mostly congruent location trials, where the color word is followed by the congruent color 75% of the time. In the remaining 48 mostly incongruent location trials, color words are followed by each of the four display colors equally often (75% incongruent). As in the original report, the model was presented four blocks of trials (384 trials total). The simulation was run twice: once with the model as normal, and once with the temporal learning mechanism lesioned. The purpose of the second run was to demonstrate context-specific contingency learning. Because the PEP model will produce a CSPC effect via *both* the contingency and temporal learning mechanisms, lesioning the latter is necessary to demonstrate the former is also contributing to the effect (and in Simulation 2, a contingency-free demonstration of context-specific temporal learning is provided). This lesion was achieved by *fixing* the response threshold, rather than allowing it to vary (see Supplementary Material).

**Table 1:** Simple context-specific proportion congruent (CSPC) design.

Color	Up (mostly congruent)				Down (mostly incongruent)			
	Red	Green	Blue	Yellow	Red	Green	Blue	Yellow
Red	9	1	1	1	3	3	3	3
Green	1	9	1	1	3	3	3	3
Blue	1	1	9	1	3	3	3	3
Yellow	1	1	1	9	3	3	3	3

**FIGURE 5:**
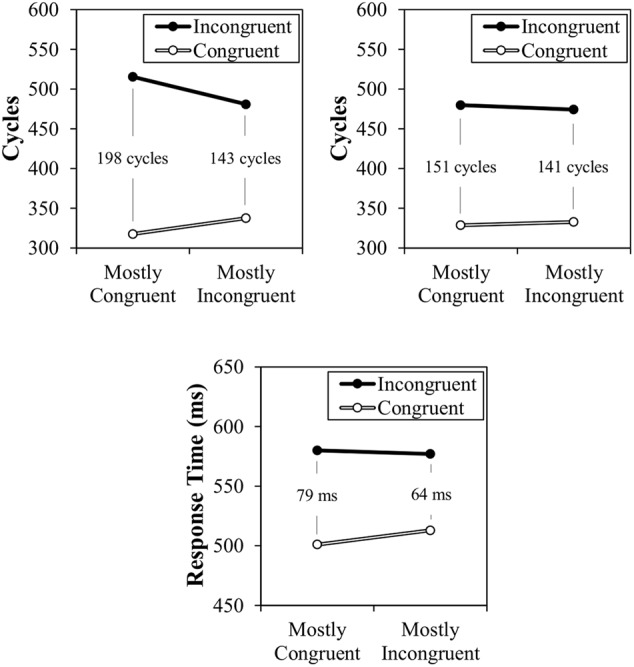
**Simulation 1 normal model cycle times (**top left**), lesioned model cycle times (**top right**), and participant response times (**bottom**)**.

The results of Simulation 1 can be observed in **Figure 5**, along with the participant data from the original report. As can be observed, a location-based CSPC effect is observed both with or without the temporal learning mechanism. However, the CSPC effect is much larger in the normal model (54 cycles) than in the model with a lesioned temporal learning mechanism (10 cycles). The latter of the two simulations confirms that the contingency learning mechanism designed for simple item-specific learning produces context-specific contingency learning without any modifications. The larger effect in the non-lesioned model demonstrates the added contribution of context-specific temporal learning (see also, Simulation 2). It is noteworthy that both the overall congruency effect and CSPC effect are larger in the modeled data than in the participant data. This might suggest that the strength of activation of the distracting word could be weakened somewhat (to be discussed in further detail in the “General Discussion”). However, the goal of the model is only to match rough qualitative fit, rather than precise quantitative fit. That is, the important point is that this simulation confirms that CSPC effects can be produced by contingency and temporal learning mechanisms. The model does not require a conflict monitor or attentional control device. It is also noteworthy that, numerically, the effect in the participant data seemed to be slightly larger for congruent trials than for incongruent trials, whereas the reverse is true in the modeled data. However, this is only an (untested) numerical trend in the participant data that does not appear to be consistent across studies (e.g., see the participant data modeled in Simulation 2).

### Simulation 2: CSPC Effect for Diagnostic Items

Simulation 2 models the CSPC effect for contingency-unbiased diagnostic items. In particular, Experiment 1 of Crump and Milliken (2009) is simulated. Diagnostic items are particularly interesting, because a CSPC effect for these items might seem strongly indicative of conflict monitoring. The present simulation demonstrates why this conclusion is too strong. The design of this experiment is presented in **Table 2**. The experiment is identical to that simulated in Simulation 1, except that there are two item types. Two items (e.g., “red” and “green”) are presented 100% with the congruent color in one location (mostly congruent context), and 100% with the incongruent color in the other location (mostly incongruent context). The remaining two items (“blue” and “yellow”) are presented equally often in the congruent and incongruent color in both location contexts. As in the original report, the model was presented four blocks of 96 trials each (384 trials total).

**Table 2:** Context-specific proportion congruent design with inducer and diagnostic items.

Color	Up (mostly congruent)				Down (mostly incongruent)			
	Red	Green	Blue	Yellow	Red	Green	Blue	Yellow
Red	12					12		
Green		12			12			
Blue			6	6			6	6
Yellow			6	6			6	6

**FIGURE 6:**
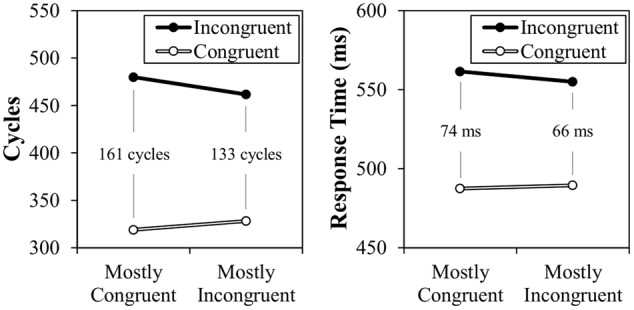
**Simulation 2 model cycle times (**left**) and participant data (**right**) for diagnostic items**.

The results of Simulation 2 are presented in **Figure 6**, along with the original participant data. As can be observed, a CSPC effect is again successfully modeled, but this time for contingency-unbiased items. This 27 cycle effect therefore demonstrates that the context-specific temporal learning mechanism does allow for the inducer items to influence the context-specific rhythm for diagnostic items. It might be again noted that the congruency effect (and, proportionately, the CSPC effect) are notably larger in the modeled data. Again, the relatively small congruency effect in the participant data likely indicates the reduced influence of the spatially- and temporally-separated word dimension.

## General Discussion

The present series of simulations demonstrated the proof-of-principle that location-based CSPC effects can result from context-specific contingency and temporal learning. Most importantly, even CSPC effects for contingency-unbiased diagnostic items can be explained by this episodic learning model. It is also critical to highlight that the model does not measure response conflict, as in the conflict monitoring model. Similarly, the PEP model does not adjust attention in response to conflict. Instead, the model maximizes performance by retrieving memories of past events in order to anticipate *what* response is likely given the stimuli presented, and *when* to respond on the basis of the same memories.

It should also be noted that the PEP model is not specifically developed to explain one narrow phenomenon. The same model, with one fixed-parameter set, also explains a range of findings outside the “cognitive control” domain, such as the power law of practice, color-word contingency learning and acquisition curves, mixing costs, and stimulus-response binding effects (Schmidt et al., 2016). It also explains a range of so-called “cognitive control” phenomena, such as item-specific PC effects, list-level PC effects for contingency-unbiased diagnostic items, congruency sequence effects in a paradigm that eliminates feature integration and contingency confounds, and asymmetric list shifting effects (Schmidt, 2013a,c, 2016a,b; Schmidt and Weissman, 2016). Including the two newly added simulations of CSPC effects, this totals 14 experiments spanning the practice, learning, binding, timing, and attentional control domains. Of particular interest, the “attentional control” phenomena are, in the PEP model, a product of the basic learning mechanisms required to simulate performance in non-conflict tasks. No conflict monitoring or attentional adjustment homunculi are needed as supplementary assumptions. The episodic account that the PEP model instantiates is therefore much more parsimonious than the conflict monitoring alternative.

Though the episodic account may be more parsimonious, it may, of course, be the case that conflict monitoring does play a role in these paradigms (e.g., in addition to the non-conflict learning biases). Indeed, the present simulations do nothing to argue against this possibility. Instead, the present simulations only demonstrate the feasibility of the simple episodic account. Some additional evidence does add further plausibility to the account proposed here, however. In addition to demonstrations of context-specific contingency learning (Mordkoff and Halterman, 2008) and context-specific temporal learning (Schmidt et al., 2014) in non-conflict tasks (i.e., where conflict monitoring is presumably impossible), King et al. (2012) reanalysed CSPC data with a linear ballistic accumulator model to contrast two competing interpretations of the effect. In particular, the conflict monitoring account suggests that CSPC effects come about because of shifts in the amount of attention to target and distracting stimuli. This implies that a change in the *drift rate* (i.e., rate of activation) for stimuli produces the CSPC effect. In contrast, the CSPC effect might result from changes in the response *threshold* (i.e., amount of evidence needed to select a response), indicative of expectancies. Their modeling results support the latter notion. This is seemingly inconsistent with the conflict monitoring hypothesis, because it is unclear how changes in attention to targets and distracters could be reflected in thresholds. King and colleagues interpret their results in terms of response caution (van Maanen et al., 2011), which is heavily related to the temporal learning account presented in the present paper (i.e., threshold adjustments resulting from expectancies), in addition to other time-based learning models (e.g., Grice, 1968; Kohfeld, 1968; Ollman and Billington, 1972; Van Duren and Sanders, 1988; Strayer and Kramer, 1994a,b; Kinoshita et al., 2011). Though these results do not rule out the possibility that conflict monitoring may play some role in the CSPC effect, like the present results, these modeling efforts add additional credence to the notion that non-attention based learning accounts are viable competing alternative explanations.

On the other hand, if attention does play some role in the CSPC effect, differences in attentional distribution in the mostly congruent and mostly incongruent contexts may originate from a factor unrelated to conflict. In particular, differences in *stimulus informativeness* in the mostly congruent and mostly incongruent conditions (Schmidt, 2014a) might lead to *contingent attentional capture* biases (Dishon-Berkovits and Algom, 2000; Jiang and Chun, 2001; Melara and Algom, 2003; Cosman and Vecera, 2014). For instance, Dishon-Berkovits and Algom manipulated the extent to which a distracting stimulus was predictive of (i.e., contingent with) the target stimulus. When a predictive contingency was present, word-word Stroop effects (i.e., both the target and distracter are words) were observed. The authors argued that attention was increased to the (predictive) distracter because it was informative for responding. However, when the distracter and targets were uncorrelated (i.e., every distracter presented equally often with all targets), the word-word Stroop effect was eliminated. They argued that, in this case, attention to the (unpredictive) distracter was reduced, thereby eliminating interference. In this way, presence of a *contingency* between distracters and targets can “capture” attention. In the original studies of Crump et al. (2006; but not in Crump and Milliken, 2009), words were predictive of the congruent color in the mostly congruent context, but unpredictive of the color in the mostly incongruent context. One might argue that this could lead to differences in contingency-driven (rather than conflict-driven) attentional capture in the two context. On the other hand, this would assume that attention to the word is adjusted *after* the word has already disappeared (i.e., when the contextual location is known). Thus, as with the conflict monitoring account, it would have to be assumed that any adjustment in processing occurs some time after initial perceptual processing. Still, if a compelling demonstration of a clear attentional contribution to the CSPC effect can be observed, additional work might focus on distinguishing between conflict monitoring and contingent attentional capture.

Before closing, it is important to note that there were some minor discrepancies between the modeled and participant data. Most notably, the model produced much larger congruency effects and, proportionately, larger CSPC effects than was observed in the participant data. Though not presented for brevity, the error congruency effect was also notably increased. Although the PEP model did produce the correct qualitative *pattern* of results (the express goal of this investigation), it is worth speculating over this quantitative difference. The model produced congruency effects and PC effects of a magnitude comparable to a standard Stroop task (i.e., color word printed in color). The reason for the much smaller effects in the participant data of CSPC experiments may originate from the fact that the distracting word is spatially- and temporally-separated from the target color. Indeed, the word is presented in a location (center) that is *never* a target location. Thus, the word likely receives less attention in such experiments, diminishing the impact of the word (Glaser and Glaser, 1982). Thus, one might reasonably argue that distracting word activation could be weakened in simulations of this sort of experiment. However, it is generally contrary to the fixed-parameter philosophy guiding development of the PEP framework to make simulation-by-simulation adjustments of parameters, so the results have been presented “as is.”

Context-specific proportion congruent effects are particularly interesting, because their existence implies that the cognitive system is able to very rapidly adapt to task-irrelevant (i.e., non-target) contextual information processed in parallel with task-relevant target stimuli. The transfer of the CSPC effect to contingency-unbiased diagnostic items is, at first glance, seemingly one of the most compelling pieces of evidence in support of the notion that conflict monitoring *must* play some role in performance. However, the current manuscript illustrates why this strong conclusion may not be warranted. Proportional and inducer/diagnostic item manipulations are typically used for the purpose of studying the effects of high versus low conflict contexts on attentional control. Unfortunately, these manipulations also introduce unintended (i.e., conflict-unrelated) regularities to the task. The human cognitive machine is particularly adept at picking up on such task regularities and using the acquired knowledge to automate performance and facilitate responding during expected events. To what extent the simple (non-conflict) episodic learning account presented in the current manuscript is a full (rather than just partial) description of CSPC effects is uncertain, but it is hoped that this report will encourage further investigation of these interesting questions.

## Author Contributions

JS wrote the code for the simulations, ran the simulations, analyzed the data, and wrote the paper alone.

## Conflict of Interest Statement

The authors declare that the research was conducted in the absence of any commercial or financial relationships that could be construed as a potential conflict of interest.

## Footnotes

1 users.ugent.be/~jaschmid/PEP/
