# Supplementary material for: Context-Specific Proportion Congruency Effects: An Episodic Learning Account and Computational Model
Source: Front Psychol. 2016 Nov 16;7:1806. doi: 10.3389/fpsyg.2016.01806 (PMC5110540; doi:10.3389/fpsyg.2016.01806)
Supplement: Supplementary file 1 [file Data_Sheet_1.pdf]

Appendix

Only one major change was made to the current version of the PEP model. In particular, the temporal learning mechanism was adapted to learn item-/context-specific temporal expectancies. Below, the math of the new mechanism is explained. Full details on the rest of the model can be found in Schmidt, De Houwer, and Rothermund (2016) or by downloading the full source code from the website of the author (users.ugent.be/~jaschmid/PEP/).

As in the previous version of the model, on each trial the response time is encoded directly into the newly-formed episode. New to the present version of the model, each episode also has a *weight* associated with the time information, which begins at .02. With each retrieval of an episode this weight weakens in the exact same way and at the exact same rate as stimulus and response weightings weaken with retrieval. Thus, newer episodes have a stronger impact on temporal expectancies than older ones, identical to the contingency learning mechanism.

At the beginning of each trial, the *pace* (i.e., expected time to respond) is computed with Formula 1,

$$pace = pace(rate) + rt(1 - rate) \qquad (\mathbf{1})$$

where *rate* is .5 and *rt* is the response time on the immediately preceding trial. Note that *pace* on the left side of the formula is the value after applying the formula, and *pace* on the right side of the formula is the value before applying the formula. The same logic applies to similar formulas below. Formula 1 therefore is simply the pace at the end of the immediately preceding trial, adjusted in response to the just-executed response time.

On each cycle of a trial, the *pace* adjusts dynamically as a result of episodic retrieval. In particular, current cycle *pace* is computed with Formula 2,

$$pace = \sum_{i=1}^{n-1} (rt_i - pace)^2 (weight_i)(activation_i - threshold) \qquad (\mathbf{2})$$

where *i* is the episode number, *n* is the current trial number, rt_i is the stored response time for episode *i*, $activation_i$ is the activation of episode *i*, and *threshold* is the threshold for episodic retrieval. Note that this formula is not computed for episodes with an activation less than the threshold (otherwise, the result would be a reverse-signed non-zero value). With this formula, each episode will push the *pace* either up or down to the extent that (a) the *rt* for that episode is different than the current *pace*, (b) the *weight* of that particular episode is strong (higher for newer episodes), and (c) the current *activation* state of the episode is high.

The above explains how the current *pace* is computed. This value influences the response *threshold* to the extent that the current *cycle* (i.e., simulated millisecond) matches the expected *pace*. In particular, on each cycle the response threshold is computed with Formula 3,

$$threshold = .25 + \frac{(cycle - pace)^2}{50{,}000} \qquad (\mathbf{3})$$

where the result of the formula is restricted between .25 and .45. Thus, with this formula, the baseline response *threshold* is .45. When the absolute difference between *cycle* and *pace* is less than 100 cycles, the *threshold* will decrease, and it can drop as low as .25 if *cycle* and *pace* are identical. This is nearly identical to the previous version of the model, except that there is one *pace* value that dynamically adapts depending on which episodes are active.

In the version of the model with temporal learning lesioned, *pace* is no longer computed. Response times and response time weightings are technically still computed by the code, but are not used. Instead, the response *threshold* is fixed at .35 (i.e., between the minimum and maximum values possible without the lesion).
